# Diversities in the Gut Microbial Patterns in Patients with Atherosclerotic Cardiovascular Diseases and Certain Heart Failure Phenotypes

**DOI:** 10.3390/biomedicines10112762

**Published:** 2022-10-31

**Authors:** Oxana M. Drapkina, German A. Ashniev, Olga A. Zlobovskaya, Adel A. Yafarova, Elena V. Dementeva, Anastasia N. Kaburova, Ivan O. Meshkov, Anna F. Sheptulina, Anton R. Kiselev, Anna V. Kontsevaya, Linar M. Zhamalov, Sergey N. Koretskiy, Mariya S. Pokrovskaya, Alexandra I. Akinshina, Anjelica V. Zagaynova, Mariia V. Lukashina, Andrey V. Kirillov, Ivan A. Abramov, Larisa R. Tolkacheva, Irina O. Bikaeva, Evgeniya V. Glazunova, German A. Shipulin, Maria M. Bobrova, Valentin V. Makarov, Anton A. Keskinov, Vladimir S. Yudin, Sergey M. Yudin

**Affiliations:** 1 National Medical Research Center for Therapy and Preventive Medicine, 101990 Moscow, Russia; 2 Centre for Strategic Planning and Management of Biomedical Health Risks of Federal Medical Biological Agency, 119121 Moscow, Russia

**Keywords:** atherosclerosis, heart failure, gut microbiome, metagenome, 16S rRNA, qPCR

## Abstract

To continue progress in the treatment of cardiovascular disease, there is a need to improve the overall understanding of the processes that contribute to the pathogenesis of cardiovascular disease (CVD). Exploring the role of gut microbiota in various heart diseases is a topic of great interest since it is not so easy to find such reliable connections despite the fact that microbiota undoubtedly affect all body systems. The present study was conducted to investigate the composition of gut microbiota in patients with atherosclerotic cardiovascular disease (ASCVD) and heart failure syndromes with reduced ejection fraction (HFrEF) and HF with preserved EF (HFpEF), and to compare these results with the microbiota of individuals without those diseases (control group). Fecal microbiota were evaluated by three methods: living organisms were determined using bacterial cultures, total DNA taxonomic composition was estimated by next generation sequencing (NGS) of 16S rRNA gene (V3–V4) and quantitative assessment of several taxa was performed using qPCR (quantitative polymerase chain reaction). Regarding the bacterial culture method, all disease groups demonstrated a decrease in abundance of *Enterococcus faecium* and *Enterococcus faecalis* in comparison to the control group. The HFrEF group was characterized by an increased abundance of *Streptococcus sanguinus* and *Streptococcus parasanguinis*. NGS analysis was conducted at the family level. No significant differences between patient’s groups were observed in alpha-diversity indices (Shannon, Faith, Pielou, Chao1, Simpson, and Strong) with the exception of the Faith index for the HFrEF and control groups. Erysipelotrichaceae were significantly increased in all three groups; Streptococcaceae and Lactobacillaceae were significantly increased in ASCVD and HFrEF groups. These observations were indirectly confirmed with the culture method: two species of Streptococcus were significantly increased in the HFrEF group and *Lactobacillus plantarum* was significantly increased in the ASCVD group. The latter observation was also confirmed with qPCR of *Lactobacillus* sp. Acidaminococcaceae and Odoribacteraceae were significantly decreased in the ASCVD and HFrEF groups. Participants from the HFpEF group showed the least difference compared to the control group in all three study methods. The patterns found expand the knowledge base on possible correlations of gut microbiota with cardiovascular diseases. The similarities and differences in conclusions obtained by the three methods of this study demonstrate the need for a comprehensive approach to the analysis of microbiota.

## 1. Introduction

Atherosclerotic cardiovascular disease (ASCVD) is an overriding concern for public health [1,2], affecting more than 500 million individuals globally and accounting for 19 million deaths annually [3]. It has been estimated that ASCVD will remain the number one killer by 2030 [4,5].

The presence of coronary artery disease (CAD) is the biggest risk factor attributed to heart failure (HF) development [6], which constitutes the final of the cardiovascular disease continuum. The classification of HF phenotypes based on the left ventricular ejection fraction (LVEF) identifies three classes of HF, namely HF with reduced EF (HFrEF) if the LVEF is less than 40%, HF with mildly reduced EF (HFmrEF) if the LVEF is 40–49%, and HF with preserved EF (HFpEF) if the LVEF is ≥50% [7]. Heart failure with preserved EF has been driven by chronic comorbidities, such as arterial hypertension, diabetes mellitus, obesity, and renal insufficiency, whilst HFrEF has been often preceded by the acute or chronic loss of cardiac cells due to coronary artery disease, myocardial infarction, various genetic mutations, myocarditis, or valvular disease [8,9].

Over the past three decades, most research has emphasized the role of gut microbiota–host interactions in human health and disease.Ground-breaking advances in high-throughput sequencingtechnologies have led to a proliferation of studies describing the impact of gut microbiota dysbiosis and certain gut-derived metabolites in a variety of CVDs, particularly in arterial hypertension [10], CAD [11], HF, and HF-related comorbidities [12]. Furthermore, accumulating evidence has suggested that gut microbial metabolites, including short chain fatty acids, trimethylamine N-oxide (TMAO), amino acid metabolites, and bile acids, are mechanistically linked to the pathogenesis of HF, and could, therefore, serve as potential therapeutic targets for HF.Gut microbiota are also implicated in the initial development of myocarditis and chemotherapy-induced cardiomyopathy through interactions with the host immune system.It has been recently explored that environmental pollutants like bisphenol A may increase doxorubicin-induced cardiotoxicity through gut microbiota deterioration and thereby worsen HF [13,14].

It has been now well-established from a variety of studies that the composition of gut microbiota in HF with reduced ejection fraction (HFrEF) significantly differs from that in a healthy state [15]. So far, the search of the literature revealed few studies with a small sample size which investigated the gut microbial composition in HFpEF in comparison to their healthy counterparts [16,17]. To date, no studies have been found that have assessed gut microbiota in patients with HFrEF/HFpEF with appropriate control groups sharing the same risk factors.

Despite notable progress in device therapy and the advent of new promising drugs, there is still ample room for improving our understanding of the processes contributing to CVD pathogenesis to develop effective preventive and therapeutic strategies [18,19,20]. Exploring the role of gut microbiota in various HF phenotypes is a topic of great interest. The differences in etiology, pathophysiology, and response rates to pharmacological therapies in patients with HFrEF and HFpEF underpin their distinct underlying molecular mechanisms which are partially tied to gut microbiota [21].

The main objective of our study is to provide a comprehensive comparative analysis of gut microbial patterns in patients with atherosclerosis (ASCVD), HFpEF, and HFrEFand the control group.

## 2. Materials and Methods

### 2.1. Cohort Description

The study complied with the Declaration of Helsinki and its later amendments, and was approved by the Institutional Ethics Committee of National Medical Research Center for Therapy and Preventive Medicine, Moscow, Russia (protocol No. 04-05/18 date: 7 June 2018, protocol No. 03-02/19 date: 11 April 2019). Prior to data collection, the participants received an explanation of the project and gave informed consent and were recruited between 2018–2020. All of the subjects were aged between 35 and 80 at the beginning of the study.

All patients underwent physical examination, collection of blood and stool samples, and 12-lead electrocardiography and transthoracic Doppler echocardiography. The evaluation of LV (left ventricular) diastolic function was based on the measurement of peak values E (early diastolic transmitral flow velocity) and e’ (early diastolic mitral annular velocity).

Confirmation of HFpEF was based on the presence of HF symptoms, left ventricular EF >50%, mitral inflow to annulus ratio (E/e’) of ≥13, and N-terminal pro-brain natriuretic peptide level of >125 pg/mL. Primary inclusion criteria for the HFrEF participants were the presence of HF symptoms and left ventricular EF <50%.With regard to ischemic etiology, the clinical course of HFmrEF is most similar to that of patients with HFrEF; therefore, we assigned patients with HFmrEF to the HFrEF group [22,23]. Eligibility criteria for the ASCVD group included angiographically proven CAD, previous myocardial infarction/transient ischemic attack, performed coronary/carotid//renal/lower extremities revascularization and an atherosclerotic aortic aneurysm. The control group comprised patients with cardiovascular risk factors such as arterial hypertension and/or dyslipidemia, but without HF and atherosclerotic lesions.

Exclusion criteria for all participants were as follows: refusal to participate in the research, body mass index (BMI) ≥ 35 kg/m^2^, psychiatric disorders, moderate or severe chronic obstructive pulmonary disease, moderate or severe asthma, acute infections or exacerbation of chronic conditions 2 weeks prior to the study enrollment, hypertrophic/dilated/restrictive cardiomyopathy, connective tissue disorders, cancer without radical treatment, pregnancy and lactation, chronic kidney disease with glomerular filtration rate <30 mL/min/1.73 m^2^, alcohol and drug addition, current smoking, inflammatory bowel diseases.

The initial sample consisted of 259 subjects, 59 of whom belonged to the HFpEF group, 50 to the HFrEF group, 100 to obstructive atherosclerosis, and the other 50 patients formed a control group. The main characteristics of the participants are presented in Table 1.

All members of the investigated groups (HFrEF, HFpEF, ASCVD, and the control group) were recruited from the National Medical Research Center for Therapy and Preventive Medicine of the Ministry of Healthcare of the Russian Federation and stayed on a standard hospital diet. The control group continued with their usual diet. All participants had not received any antibiotics or laxatives in the month prior to the study.

### 2.2. Blood and Stool Sample Collection and Preparation

Blood sampling for the biochemical analysis and determination of NT-proBNP (N-terminal prohormone of brain natriuretic peptide) was carried out from the cubital vein in the morning on an empty stomach. The concentration of NT-proBNP was assessed using a Multiscan FC photometer (Thermofisher Scientific, Waltham, MA, USA) only in HFpEF group.

Fecal samples were obtained from the National Medical Research Center for Therapy and Preventive Medicine of the Ministry of Healthcare of the Russian Federation. Human stool samples were donated by patients and healthy volunteers who signed informed consent in accordance with the local Ethic Committee (protocol No. 04-05/18 date: 7 June 2018, protocol No. 03-02/19 date: 11 April 2019).

Immediately after delivery, the stool samples were frozen at −80 °C until use. After thawing, the stool aliquots were separated into groups for two independent sets of DNA extraction. Each aliquot was obtained by taking a 200 mg scraping from each individual fecal sample.

### 2.3. Microbial Identification by MALDI-TOF-MS

For relevant analysis the control groups were selected according to the characteristics of the patients in the selected sample groups: the criteria for the control group of samples included the restrictions for differences in the mean age and BMI, which had to be no more than 15 years and 3 kg/m^2^ respectively between each investigated group and the control group (this condition was met for all variants of the analysis below). The characteristics of the resulting groups are presented in Table 2.

All fecal samples were used for cultivation of various groups of microorganisms. Of each sample, 1 g aliquots were homogenized for 1 min and were resuspended in 9 mL of sterile 0.9% saline solution. From the obtained solution, a series of subsequent dilutions of the suspension in saline was prepared as follows: 1 mL was taken from the initial solution with a dispenser and placed in a second test tube with saline until a dilution of 10^−9^ was obtained. A suspensions aliquot was cultivated in a set of selective media and incubated at 37 °C for 24–48 h in anaerobic and aerobic conditions, respectively. At the end of the incubation time, each type of microbial colony was characterized macroscopically and microscopically and identified by mass spectrometry with MALDI-TOF MS, using the MALDI Biotyper system with Microflex (Bruker Daltonics Inc., Billerica, MA, USA). For this procedure, the direct method for extracting proteins from microorganisms in plaque was performed by applying a small amount of the colony on a plaque of the MALDI in duplicate, allowing it to dry at room temperature. Subsequently, 1 μL of formic acid was added to the colonies, allowed to dry, and then 1 μL of the matrix solution (alpha-cyano-4-hydroxycinnamic acid) was added to extract proteins, mainly ribosomal, present in high concentrations, and again allowed to dry at room temperature. The mass spectra were analyzed within a range of 2000 to 20,000 *m*/*z*. The MALDI Biotyper version 3.0 library and the MALDI Biotyper version 3.0 software were used for identification considering the scores 1.5–1.7 for the genus level and scores >2 for the species level.

### 2.4. DNA Extraction and Preparation of DNA Libraries and Sequencing

DNA extraction was conducted for samples from patients according to Table 2.

DNA was extracted using either QIAamp^®^ Fast DNA Stool Mini (Qiagen, Germany) or QIAamp^®^ PowerFecal Kit (Qiagen, Germany) according to the manufacturer’s instructions. A dsDNA HS Assay Kit and Qubit^®^ 4.0 fluorometer (Thermo Fisher Scientific, the USA) were used to measure the DNA concentration, and the quality of isolated DNA was analyzed by electrophoresis in 1% agarose gel.

DNA libraries were prepared using polymerase chain reaction (PCR) amplification with gene-specific primers for the V3–V4 regions of 16S rRNA. Processes, such as DNA fragmentation, repair, adapter ligation, and unbiased PCR amplification, were performed strictly in accordance with Illumina instructions. The quality of the prepared libraries was analyzed on the Bioanalyzer 2100 (Agilent Technologies, Santa Clara, CA, USA) using the High Sensitive DNA kit (Agilent Technologies, USA). A Qubit dsDNA HS Assay Kit and Qubit^®^ 4.0 fluorometer were used to measure the DNA concentration in each sample. The quality-qualified libraries were pooled in a 0.2-mL PCR tube. A MiSeq Reagent Kit V2 Nano was used to prepare the DNA for sequencing. Finally, the samples were sequenced using the Illumina MiSeq platform (Illumina, San Diego, CA, USA) according to the manufacturer’s protocol using reagents for double-ended reading and a read length of at least 250 b.p. The amount of PhiX Control v3 was not less than 1%.

### 2.5. Metagenome Data Processing and Analysis

Reads were analyzed using the Quantitative Insights in Microbial Ecology 2 (QIIME2), v 2022.2.0 [24]. Primers flanking the V3–V4 region of 16S rRNA for all cohorts were removed from fast reads using the cut adapt plugin implemented in QIIME2 [25]. After that, reads were trimmed, filtered, and merged via DADA2 [26], resulting in a table of all amplicon sequence variants (ASVs) presented within a set of samples.

For relevant analysis, the control groups were also selected according to the characteristics of the patients in the selected sample groups. The characteristics of the resulting groups are presented in Table 3.

**Figure 1:**
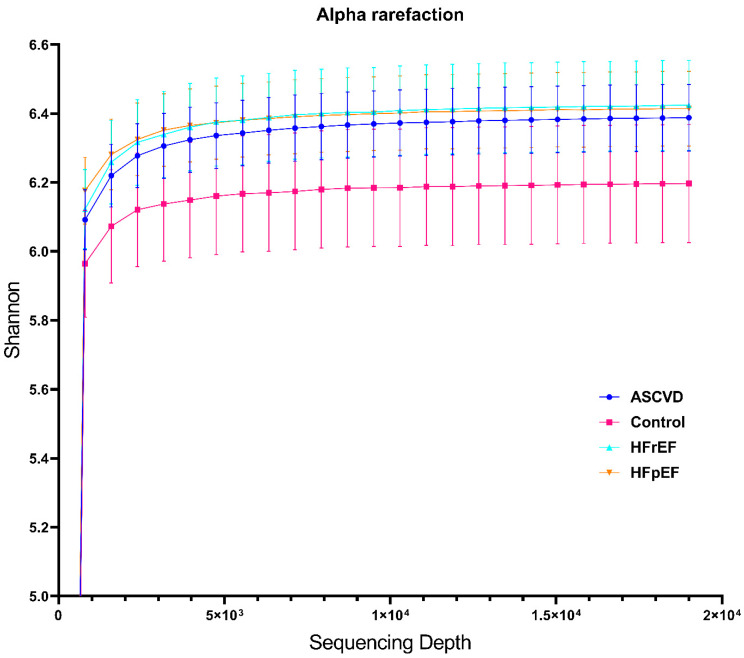
Rarefaction curves showing Shannon diversity index at various sequencing depths. Curves reached a plateau at approximately 5000 sequences and showed a similar manner of diversity enrichment for every group except the healthy cohort. The evaluation for every nosology started at the sequencing depth with a zero diversity index.

Next, we generated multiple sequence alignment using MAFFT [27] and built a phylogenetic tree via the FastTree plugin [28]. The generated tree was midpointed at the largest root–leaf distance. Taxonomy assignment to ASVs was performed with a Naive-Bayes classifier (scikit-learn library) trained on SILVA v.138.99 and RDP v.11.5 reference 16S rRNA databases, taxonomy data at the family level were included in the analysis. Further, we calculated six alpha-diversity indices (Shannon, Faith, Pielou, Chao1, Simpson, and Strong) to access sample richness and/or evenness. Rarefaction curves showed an asymptotic behavior by group and thus indicated the appropriate 19k sampling depth for analysis (Figure 1).

UniFrac distance [29] was developed by Catherine Lozupone and Rob Knight and used for assessing beta-diversity in order to determine whether sample communities are significantly different. The bacterial dissimilarity was measured by unweighted UniFrac, as a qualitative measurement, and weighted UniFrac, as a quantitative measurement. We assumed the independency of the calculated dissimilarity between analyzed samples. The obtained matrices were used for principal coordinates analysis (PCoA), linear discriminant analysis (LDA), and Mann–Whitney Testing (see Section 2.7 Statistical analysis).

### 2.6. Real-Time qPCR Quantification of Bacterial DNA

#### 2.6.1. Study Group

For qPCR analysis, samples from all investigated groups (HFrEF, HFpEF, ASCVD) were selected from the total amount of previously extracted samples. Samples from the investigated groups were chosen according to their DNA concentration and total DNA yield required for the correct setting of the study. Then the control group was selected according to the characteristics of the patients in the selected sample groups: the criteria for the control group of samples included the restrictions for differences in the mean age and BMI, as well as sufficient DNA concentration and total yield for all qPCR reactions.

The qPCR study population included 64 patients diagnosed with ASCVD, 58 patients with CHF disease (n = 33 for HFrEF and n = 25 for HFpEF), and 38 volunteers from the control group. As previously mentioned, two different fecal DNA extraction methods were used in this study: QIAamp Fast DNA Stool Mini Kit (FS) and QIAamp PowerFecal DNA Kit (PF); hence, sampling for both methods was performed. For some individual fecal samples, DNA was extracted with both kits.

The characteristics of the resulting groups are presented in Table 4 and Table 5.

#### 2.6.2. PCR Reaction

The nonspecific total bacterial DNA yield was evaluated by amplification of the V4 region using universal primers. The DNA amount of the families Enterobacteriaceae, Lactobacillaceae, Christensenellaceae; genera *Bacteroides* sp., *Bifidobacterium* sp., *Odoribacter* sp., *Oscillibacter* sp.; species *Enterococcus faecalis*, *Faecalibacterium prausnitzii* was evaluated using corresponding primers and TaqMan probes (Table S1). All primer sets were evaluated for their specificity and sensitivity in a broad range quantification real-time PCR using strains (Federal Medical-Biological Agency, Russia) and purified bacterial DNA from the Leibniz collection (DSMZ, Leibniz, Germany). Standard curves were obtained for each primer set using serial 1:10 dilutions (from 10^6^ to 1 copy of 16S rRNA gene in reaction) of measured purified bacterial DNA from the aforementioned collections. The corresponding slope and interception factors were calculated for each standard curve, using standard samples’ concentrations expressed in gene copies/μL.

Prior to the qPCR experiment, the DNA concentration of the samples was determined fluorometrically on the Qubit^®^ 4.0 Fluorometer (Thermo Fisher Scientific, Waltham, MA, USA) using the Qubit^®^ dsDNA BR Assay Kit. Each sample was then diluted in sterile purified water to a concentration of 1.0 ng/µL. Those diluted aliquots were used in real-time qPCR assays and were stored at 4 °C between plate runs to preserve the samples from degradation.

All qPCR reactions were carried out using the 5X PCR Master Mix (the Center for Strategic Planning, Moscow, Russia) containing hot-start Taq-polymerase and 17.5 mM MgCl_2_, in the conditions recommended by the manufacturer, with 400 nM of each primer and TaqMan probe in a volume of 25 μL. Additionally, 96-well optical-grade PCR plates sealed with optical sealing tape (Bio-Rad, Hercules, CA, USA) were used. Sterile water served as the no template control. Each plate contained reactions of one qPCR assay to minimize threshold-dependent biases and all PCR runs contained a standard sample which was used to set a correct threshold in each plate (10% of control sample end fluorescence).

The PCR program was: 95 °C—15 min, 45 cycles: (95 °C—30 s, 60 °C *—30 s, 72 °C—30 s) on CFX96 Touch™ (Bio-Rad Laboratories, Inc., Hercules, CA, USA).

#### 2.6.3. Bacterial DNA Quantification

Standard curves for each primer set and corresponding slope and interception factors described previously were used to determine DNA sample concentration using Formula (1):

C = 10^(Ct-int)/slope^ (1)

where C is the initial target concentration in gene copies per μL, Ct is the cycle threshold for the sample, ‘int’ stands for intercept.

Given the fact that 16S rRNA genes are present in varying numbers in bacterial species, the obtained OTU or total bacterial concentrations were calculated using copy number data for the 16S rRNA sequences provided by the National Library of Medicine (NCBI). In the next step, for use in statistical analysis and calculations, OTU were normalized to the total bacterial concentration for each individual sample (thus relative abundance was calculated) and then all OTU concentrations were adjusted to a value at a total bacterial count of 10^7^ per μL.

### 2.7. Statistical Analysis

#### 2.7.1. Software

All calculations were performed using the R programming language (v 4.2.1) and the data processing environment RStudio 3 February 2022 (build 492).

#### 2.7.2. Data Conversion

(1) Raw clinical data contained sample ids, morphometric and demographic indicators, cardiovascular system state, and hematological and biochemical parameters.
  The table included binary and quantitative values which were transformed in accordance with the following formulas (Formulas (2)–(3)):
  (2) $$z_{ij}=\frac{x_{ij}-Med_{j}}{IQR_{j}}$$
  (3) $$v_{ij}=\frac{1}{1+\exp\left(-z_{ij}\right)}$$
  where $x_{ij}$—initial value of the quantitative indicator presented at the *i*-th row, in the *j*-th column, $Med_{j}$—median value of *j*-th column, $IQR_{j}$—interquartile range of the *j*-th column, $\;v_{ij}$—converted value.
  Taking into account the influence of outliers presented in the data often leads to the misleading interpretations and wrong conclusions. This explanation holds true for all further conversions during multivariate analysis.
(2) MALDI-TOF mass spectra profiles of every sample were transformed in accordance with the following rule:
  - all zero elements were retained,
  - nonzero elements were converted by formula (Formula (4)):
    (4) $$v_{ij}=\log_{10}x_{ij}$$
    where $x_{ij}$—original non-zero value located in the *i*-th row, in the *j*-th column,$\;v_{ij}$—converted nonzero value.
(3) PCR tables included sample ids columns and corresponding relative DNA-concentration values, which also were processed in accordance with the following rule (Formula (5)):
  (5) $$v_{ij}=\tanh\left(\frac{x_{ij}-Med_{j}}{IQR_{j}}\right)$$
  where $x_{ij}$—the initial value of the quantitative indicator, located on the *i*-th line, in the *j*-th column, $Med_{j}$—median value of the *j*-th column, $IQR_{j}$—interquartile range of the *j*-th column, tanh—hyperbolic tangent, $\;v_{ij}$—converted value.
(4) We also provided custom normalization for 16S rRNA NGS data. During the bioinformatics read processing, we obtained a table which included sample ids and counts for every taxonomical string. Despite the validity of the taxonomical ratio provided by rarefaction curves, low abundant family groups (Brachyspiraceae, Burkholderiaceae, Dermatophilaceae, etc.) commonly reveal abundancy sampling biases. In order to minimize sampling effects, we ranged low abundant taxonomy groups in accordance with the following rule. All values of ASV-frequency table below 31 were changed to 0, and those from 31 to 99 were replaced by 75. Furthermore, values from 100 to 149 were changed to 125. Counts outside the specified ranges remained unchanged.

#### 2.7.3. Data Filtering and Exploratory Analysis

From the studied data we chose observations of the control and the comparison groups, and formed two multivariate samples. The intergroup comparison showed considerable difference in age and body mass index (BMI) between initial groups while the difference between the average sample values of age and BMI should not exceed 15 years and 3 kg/m^2^, respectively. Thus samples were filtered to bring those parameters closer to each other.

If observed intersample differences did not match the required parameters, we used 3 types of algorithms for sample convergence. The compared samples are denoted as $A\in \left\{a_{1},\;a_{2},\ldots ,\;a_{n}\right\}$ и $B\in \left\{b_{1},\;b_{2},\ldots ,\;b_{m}\right\}$, where n and m are sizes and $T_{j}$ и $T_{k}$—required differences in terms of j and k, respectively.

Algorithm I was applied in the case of different sample sizes and tangible difference between the sample means d. For example, sample A was larger than sample B, i.e., *n*> *m*.

The difference between samples averages was calculated as (Formula (6)):

(6) $$d_{j}=\bar{A}-\bar{B}=\left(\frac{\sum_{i=1}^{n}a_{ij}}{n}\right)-\left(\frac{\sum_{i=1}^{m}b_{ij}}{m}\right)$$

If $d_{j}>0$, sample A was truncated by maximum value and by minimum value if $\;d_{j}<0$. Then the difference was calculated again and compared with the required difference, if necessary, the removal of the maximum or minimum value was repeated up to the step until $d_{j}$ became less or equal to $T_{j}$.

Algorithm II was applied in the case of equal sample sizes and tangible difference between the sample means d, i.e., *n* = *m*. Again, the difference between samples averages was calculated as (Formula (7)):

(7) $$d_{j}=\bar{A}-\bar{B}=\frac{\sum_{i=1}^{n}a_{ij}-\sum_{i=1}^{n}b_{ij}}{n}$$

If $d_{j}>0$, sample A was truncated by minimum value and sample B by maximum value. However, if $\;d_{j}<0$, sample A was truncated by maximum value and sample B by minimum value. The process was repeated up to the step when $\;d_{j}$ became less or equal to $T_{j}$ Algorithm III was applied in the case of different sample sizes and observed tangible difference between the sample means *d* for both indicators *j* and *k* ($d_{j}>T_{j}$; $d_{k}>T_{k}$), i.e., *n* > *m*. At each step,$\;d_{j}\;$ and $d_{k}$ were calculated as following (Formulas (8)–(9)):

(8) $$d_{j}=\left|\left(\frac{\sum_{i=1}^{n}a_{ij}}{n}\right)-\left(\frac{\sum_{i=1}^{m}b_{ij}}{m}\right)\right|$$

(9) $$d_{k}=\left|\left(\frac{\sum_{i=1}^{n}a_{ik}}{n}\right)-\left(\frac{\sum_{i=1}^{m\;}b_{ik}}{m}\right)\right|$$

Samples were filtered in a two-dimensional space with a preliminary standard setting of *j* and *k* values:

(10) $$v_{ij}=\frac{x_{ij}-Mean_{j}}{Sd_{j}}$$

where $x_{ij}$—initial value of *j*-th variable, $Mean_{j}$—sample mean of variable *j*, $Sd_{j}$—sample standard deviation of variable *j*, $\;v_{ij}$—converted value.

Among the observations of a smaller sample, there is one that satisfies the following condition (Formula (11)):

(11) $$v_{medoid}=\arg min_{b\in \left\{b_{1},\;b_{2},\ldots ,\;b_{m}\right\}}\left(\overset{m}{\underset{i=1}{\sum }}eucl(b,v_{i}\right))$$

The expression $eucl\left(b,\;v_{i}\right)$ denotes the Euclidean distance in the space of the converted age and BMI values, i.e., variables j and k. The expression “arg min” denotes the value at which the parenthesized expression reaches its minimum. In other words, a sample of a smaller size contains a medoid, an observation whose difference from all other observations of the same sample is minimal. Then, between the found medoid and all observations of the larger sample $A\in \left\{a_{1},\;a_{2},\ldots ,\;a_{n}\right\}$ we calculated the Euclidean distance. Finally, the observation with the largest distance from the found medoid was excluded from sample A.

The exclusion of observations from a larger size sample continued until the differences between the sample means (in terms of *j* and *k*) decreased.

The filtering technique was applied to the following samples pairs:

1. Control group and ASCVD
2. Control group and HFpEF
3. Control group and HFrEF

After the filtering, the results of qPCR, 16S rRNA sequencing and MALDI-TOF data processing were used for PCA analysis in combination with linear discriminant analysis (LDA).

The principal components’ values and total scatter proportion of the original data were calculated using the prcomp function with default parameters. The first few principal components were identified and chosen for further analysis. Chosen components explained about a third of the total data dispersion in a multidimensional space. According to this criterion, no more than 10 main components were to be singled out for the subsequent discriminant analysis.

Discriminant analysis was performed implementing the LDA function with the default parameters. ThePCA and LDA methods’ combination allowed us to identify variables which underlie statistically significant differences between samples. Revealed variables were subsequently statistically investigated. Using LDA, we constructed the best separative hyperplane and drew a discriminant axis in the direction of the maximum intergroup data variability. The discriminant axis is a linear combination of the main components, which represent the original prevalence indicators of microorganisms. The correlation coefficients were estimated using the Spearman method.

For the further statistical analysis, we considered only those correlation coefficients with the values equal in absolute value to 0.3 (rho≥0.3).

#### 2.7.4. Statistical Analysis

For the statistical analysis we used previously combined and filtered samples pairs.

The intergroup analysis included Mann–Whitney Testing. The effect size was estimated by the formula (Formula (12)):

(12) $$\eta^{2}=\frac{H-k+1}{n-k}$$

where H—Kruskell–Wallis estimator, k—number of compared groups, n—total number of observations for both samples. The *p*-value calculation was carried out using the Monte Carlo algorithm with 1 million simulations in each calculation.

#### 2.7.5. Statistical Analysis of Clinical Diagnostic Data

The clinical data analysis did not include preliminary exploratory analysis.

For the statistical analysis, we used previously combined and filtered sample pairs. For the quantitative indicators we used Mann–Whitney Testing and a Fisher’s Testing modification for qualitative indicators. The *p*-value calculation was carried out using the Monte Carlo algorithm with 1 million simulations in each calculation.

The effect size was calculated using the aforementioned formula (for the quantitative indicators). However, for the binary indicators, the mean square contingency coefficient φ was estimated.

To control the false discovery rate, all obtained *p*-values were corrected according to the Benjamini–Yekutieli procedure.

## 3. Results

### 3.1. Bacterial Culturation Study

Table 6 provides a wide range of statistically significant associationsobtained from the MALDI-TOF MS analysis.

More than 300 species were identified in bacterial cultures for all groups. Their presence and quantity in the investigated groups of diseases differed significantly, but there were some taxa for which the decline in representation correlates with all three groups. For instance, the decrease in abundance of *Enterococcus faecium* had the most pronounced effect for the ASCVD and HFpEF groups in both cases, and was also found in the HFrEF group, but in the case of the latter, the most significant effect was a decreased abundance of Enterococcus faecalis.

**Figure 2:**
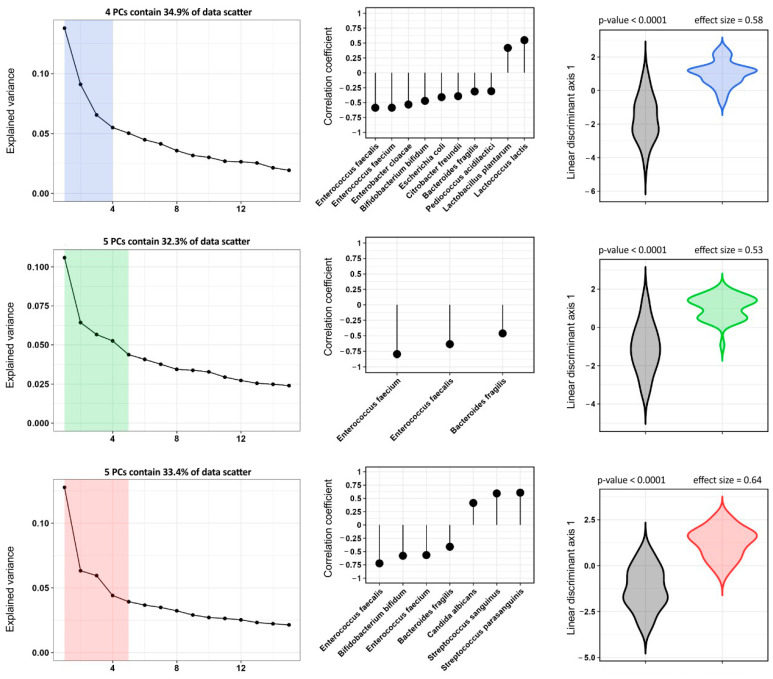
Results of PCA data processing (MALDI-TOF MS), the established correlation between the best discriminant axis and initial indicators, the distribution of the observed group values across the best discriminant axis. The top row of graphs is a comparison of patients of the control group and patients with atherosclerosis, the middle row is a comparison of patients of the control group and patients with HFpEF patients, the bottom row of graphs is a comparison of patients of the control group and patients with HFrEF patients.

Interestingly, for ASCVD, there were no taxa that increased in abundance with a size effect more than 0.1: the only two significant increases were seen in *Lactobacillus plantarum* (*p* < 0.01, size effect 0.09) and *Lactococcus lactis* (*p* < 0.01, size effect 0.07) (Figure 2).

Participants from the HFpEF showed the least difference compared to the control group, demonstrating only relatively decreased abundance of three species.

### 3.2. Metagenome Data

As the samples were obtained by two different extraction kits (PF and FS) of the same manufacturer, at the first step the method-dependent effect on qPCR data using principal component analysis (PCA) and linear discriminant analysis (LDA) was investigated. After using dimensionality reduction, a cluster box plot for combined samples was obtained. Profiles of each method in the plots indicate that the choice of DNA extraction method did not affect the results of the NGS study, so samples were combined for further statistical calculations and analysis.

**Figure 3:**
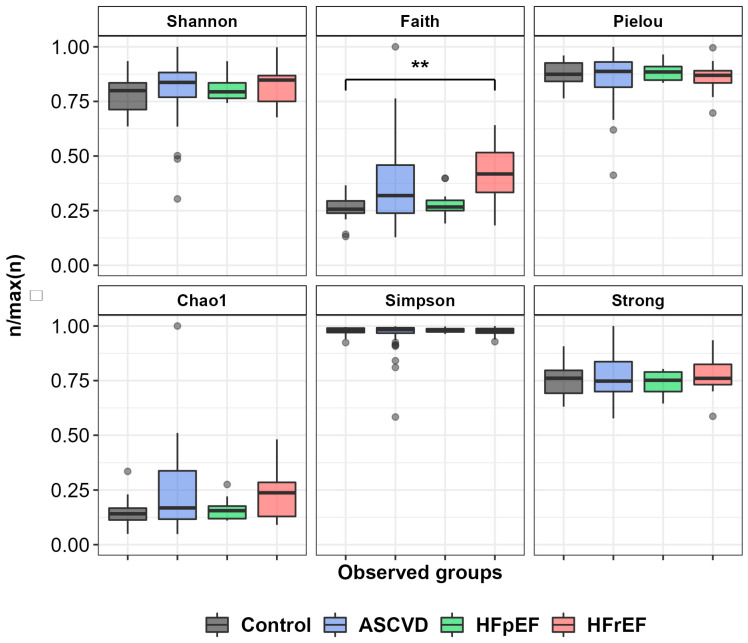
Analysis of alpha diversity of samples’ microbiome. ** indicates *p* < 0.01.

Six alpha-diversity indices (Shannon, Faith, Pielou, Chao1, Simpson, and Strong) were analyzed for all groups of samples to estimate richness and evenness of gut microbiota composition. No significant differences between patient’s groups were observed (Figure 3) with the exception of the Faith index for the HFrEF and control groups (*p* < 0.01).

The results obtained at the family level for statistically significant taxa found in both databases (RDP and Silva) with an effect size more than 0.1 are shown in Table 7.

Gut microbiota composition in the ASCVD group was associated with several taxa, with the biggest effect size in the families Erysipelotrichaceae and Streptococcaceae (increased compared to the control group) and Sutterellaceae (decreased). Changes in the families Clostridiales_Incertae Sedis XIII (*p* <0.01, size effect 0.13) or Coriobacteriaceae (*p* < 0.05, size effect 0.08) were also statistically significant, but were met in very low numbers for both control and disease samples, thus forcing one to be more careful in assessing their clinical significance.

**Figure 4:**
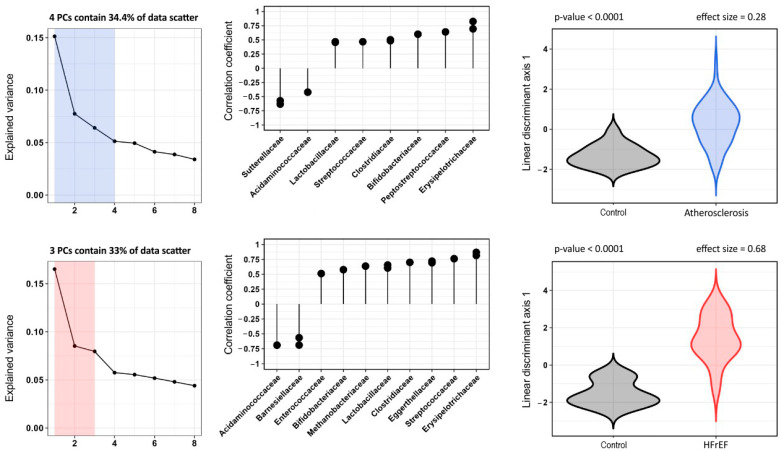
Results of PCA data processing (metagenome), the established correlation between the best discriminant axis and initial indicators, the distribution of the observed group values across the best discriminant axis. The top row of graphs is a comparison of patients of the control group and patients with atherosclerosis, the bottom row of graphs is a comparison of patients of the control group and patients with HFrEF patients.

The most pronounced diversities in the composition of gut microbiota were found in the HFrEF group, with more than ten families found statistically significant in both databases. Curiously, the family Sutterellaceae (*p* < 0.01, size effect 0.44) was found only in the RDP database, probably because of its low abundance in both states.The greatest changes were again seen in the families Streptococcaceae and in Erysipelotrichaceae (increased in HFrEF group) and in the family Acidaminococcaceae (decreased, as was also seen in ASCVD group) (Figure 4).

Interestingly, participants from the HFpEF showed the least difference compared to the control group, demonstrating only relatively increased prevalence of family Erysipelotrichaceae.

### 3.3. Real-Time qPCR Analysis

As the samples were obtained by two different extraction kits (PF and FS) of the same manufacturer, at the first step, the method-dependent effect on qPCR data using principal component analysis (PCA) and linear discriminant analysis (LDA) was investigated. After using dimensionality reduction, cluster box plots were obtained for all experimental and control groups (Figure S1). Profiles of each method in the plots indicate that the choice of DNA extraction method affected the results of the qPCR study and could change the prevalence of some microorganisms, so combining samples for further statistical calculations was not possible and the analysis proceeded independently for both methods (Figure S2).

At the next step, obtained OTU concentrations from qPCR analysis for two extraction methods and each pair disease-control were used for Mann–Whitney U test with Benjamini–Yekutieli correction for multiple testing (including clinical data, NGS, and bacterial culture results).

For the FS extraction method, the microbiota of ASCVD patients compared to healthy subjects were characterized by a significantly higher abundance in Lactobacillaceae (corrected *p*-value 0.004, size effect 0.22). Taxa Enterobacteriaceae, Christensenellaceae, *Bacteroides* sp., *Bifidobacterium* sp., *Odoribacter* sp., *Oscillibacter* sp., *Enterococcus faecalis*, *Faecalibacterium prausnitzii* did not show significant differences between the two groups.

Before *p*-value correction, some differences between other groups and the control group were also observed: the microbiota of HFrEF patients exhibited a lower abundance in *F. prausnitzii* (*p*-value before correction 0.006, size effect 0.14) and in *Bacteroides* sp. (*p*-value before correction 0.024, size effect 0.09) and a slightly higher abundance in Lactobacillaceae (*p*-value before correction 0.025, size effect 0.09). HFpEF patients had a higher abundance in *Oscillibacter* sp. (*p*-value before correction 0.006, size effect 0.16); however, after Benjamini–Yekutieli correction all these values turned insignificant.

## 4. Discussion

### 4.1. Comparison of qPCR Data between Extraction Methods and with the NGS Data

Relative abundances of the families Enterobacteriaceae, Lactobacillaceae, Christensenellaceae; genera *Bacteroides* sp., *Bifidobacterium* sp., *Odoribacter* sp., *Oscillibacter* sp.; species *Enterococcus faecalis*, *Faecalibacterium prausnitzii* were evaluated using qPCR for four groups: ASCVD, HFrEF, HFpEF, and the control.

The qPCR data for each of the four studied groups were quite clearly separated on the principal component plot depending on the isolation method used, which allowed us to consider the quantitative assessment by the qPCR method in this case to be quite accurate. A similar difference was not noted for NGS, which may be explained by the less precise quantification that this method is capable of, due to the different amplification efficiencies of different targets by degenerate primers. Due to the observed difference between the isolation methods, the results for the qPCR for each method were compared with the NGS results separately, as well as among themselves.

Analysis and comparison of data for both types of study (NGS, qPCR) showed that for ASCVD samples the number of Lactobacillaceae was statistically higher than in the control group (*p* < 0.01 for qPCR of FS samples and *p* < 0.01 for NGS). This was observed in the case of isolation with the FS method, but not with PF, which can be explained by the fact that different patient samples were used for the two isolation methods. However, as a similar observation regarding an increased content of the Lactobacillaceae family, DNA was obtained in the analysis with the NGS method and in bacterial culture, this allowed us to make a more confident assumption about its possible correlation with this disease.

For other taxa, the difference was not statistically significant (*p* > 0.05) both for genera or species in qPCR and their corresponding families in NGS and for matching families in qPCR and NGS. Interestingly, qPCR of genera *Bifidobacterium* sp. and *Odoribacter* sp., did not show statistical significance between groups, while the difference for the families Odoribacteraceae and Bifidobacteriaceae was significant (*p* < 0.01) or conditionally significant (0.01 < *p* < 0.05) according to NGS data. The same goes for HFrEF samples, where families Bifidobacteriaceae, Enterococcaceae, and Odoribacteraceae show some conditionally significant differences (0.01 < *p* < 0.05) according to NGS data—but qPCR does not detect them for *Bifidobacterium* sp., *Enterococcus faecalis*, or *Odoribacter* sp.

For the family Lactobacillaceae in HFrEF samples, where NGS is more confident (*p* < 0.01) there is also a hint in qPCR data for the FS method (*p* < 0.05 prior to correction). For the rest of the taxa for the HFrEF samples as well as for all the taxa of HFpEF samples, results of qPCR and NGS both agree on the absence of significant difference with the control group.

Some discrepancies in qPCR and NGS results could be explained by several reasons. First, only specific taxa from the families were selected for the qPCR study, so probably other genera and species contributed to the differences observed. Secondly, in the qPCR study, the controls were closer matched with the disease groups in BMI and sex ratio parameters than in the NGS study, which can explain the smaller number of observed statistically significant differences in the qPCR results. It should also be noted that the identification of reads as Odoribacteraceae was carried out only using the RDP Ribosomal Database library and there were no matches with SILVA, which may also be the reason for the discrepancy between the conclusions from qPCR data.

Curiously, despite the absence of clear differences between the samples of patients and healthy individuals for each particular taxon, the combination all PCR taxa for the principal component method and linear discriminant analysis managed to reliably separate healthy controls from patients with atherosclerosis for both extraction methods (*p* < 0.01 for PF and *p* < 0.05 for FS, see Figure S2).

### 4.2. Analysis of NGS Data and Trends in Prior International Studies

In this research, we determined the differences in gut microbiota composition between ASCVD, HFpEF, and HFrEF patients and the controls. Interestingly, all investigated groups demonstrated an increase in the Erysipelotrichaceaefamily. One unanticipated finding was that the composition of HFpEF showed the least difference compared to the control group, demonstrating relatively increased prevalence of family Erysipelotrichaceae, while theGUMPTION Study (Gut Microbiota in Heart Failure Patients With Preserved Ejection Fraction) determined the differences in gut microbiota between HFpEF patients and controls. According to this study, the abundance of *Butyricicoccus, Sutterella, Lachnospira,* and *Ruminiclostridium* in the HFpEF group was lower than that in the control group, while the abundance of *Enterococcus* and *Lactobacillus* was higher [16]. Another study demonstrated a depletion of short chain fatty acids producing bacteria, particularly *Ruminococcus* [17], which has not been conclusively proven in our study.

Gut microbial composition both in ASCVD and HFrEF groups negatively correlated with an abundance of specific butyrate-producing phyla including family Odoribacteraceae, which correlates with published data [30]. The gut microbial feature of ASCVD group increased in the abundance of Clostridiaceae and Peptostreptococcaceae which were positively associated with TMAO production in humans. This finding also accords with earlier observations, which showed that uncharacterized members of Erysipelotrichaceaeand Lachnospiraceae families in the gut are positively correlated with total cholesterol and low-density lipoprotein cholesterol levels [31]. Furthermore, it was shown that increasing members of the Erysipelotrichaceae family positively correlated with lipopolysaccharide (LPS)-induced cytokine secretion: TNF-α GlcNAc-6-P: N- acetylglucosamine-6-phosphate, IL-1β [32]. Lueddle et al. (2017) found that certain HF patients with extremely reduced LVEF (22.3 ± 2.85%) showed significant reductions in unidentified Erysipelotrichaceae and Ruminococcaceae spp populations which differs from our findings [33]. This result may be explained by the fact that most participants of our HFrEF group had a I-II NYHA functional class and higher LVEF (median LVEF 34%). However, the significant decrease of Coriobacteriaceae inHFrEFobservedin this study corroborates our findings [33]. According to the literature, the increase of members of the Porphyromonadaceae family correlated with acute ischemic stroke and a high level of SCFA, which lead to the increase of inflammatory response [34].

The gut microbial feature of HFrEF group increased in the abundance of Coriobacteriaceae which were associated with increasing of TMAO production. TMAO directly contributes to platelet hyperreactivity and enhances thrombosis potential for patients with stroke and heart failure, particularly for the HFrEF group. This positive correlation between gut microbiota and the risk of thrombosis was also shown in vivo [35].

In HFrEF, we also observed an increased abundance of strict anaerobes Methanobacteriaceae which can produce methane by reducing carbon dioxide and molecular hydrogen. These results reflect those of Fadeeva M.V. et al. (2020) who also found that an increase in the proportion of Methanobacteriaceae in heart failure patients with systolic dysfunction [36]. According to the results of 16S RNA sequencing analysis, gut microbiota composition in ASCVD group was associated with relatively decreased Sutterellaceae, in turn, the HFrEF group was associated with a relatively increased abundance of the same family at the same time with the increasing abundance of Erysipelotrichaceae. Negative correlation of gut microbiota pattern (Caproiciproducens, Sutterellaceae and Erysipelotrichaceae) with blood lipid profiles, inflammation cytokine, and myocardial enzyme was shown in an in vivo experiment. These results suggest that the modulation of gut microbiota might be one mechanism to prevent and treat cardiovascular diseases after acute cardiovascular events, which characterized the HFrEF group [37].

Intriguingly, both the ASCVD and HFrEF groups demonstrated increased prevalence ofLactobacillaceae, which was not reported in earlier observations in the setting of such conditions. Furthermore, both ASCVD and HFrEF groups demonstrated an increase in the Streptococcaceae family. It is difficult to explain this result, but it might be related to the fact that most patients from these groups took antiplatelet agents and/or anticoagulants. In order to reduce the risk of gastrointestinal bleeding, they also received proton pump inhibitors which were associated with an increase in the Lactobacillales order, and in particular the family Streptococcaceae [38]. However, an increase in levels of Lactobacillaceae members has been shown in patients with acute ischemic stroke [34]. Increased prevalence ofLactobacillaceaewas correlated with high levels of systematic inflammatory response, associated with an increase of LPS levels. Decreased prevalence ofBarnesiellaceae and Acidaminococcaceae, which was shown inboth the ASCVD and HFrEF groups, associated with decreasing of synthesis of SCFA (valeric and propionic acids) and increasing of TMAO levels, lead to an increase in the level of proatherogenic lipoproteins in the blood (cholesterol metabolism). It is assumed that these microbial communities can influence the formation of atherosclerotic plaques [39].

In the HFrEF group, wealso observed a relatively increased abundance of Enterococcaceaewhich are conditional pathogens, causing infections such as urinary tract infections, purulent abdominal infections, sepsis, endocarditis, and diarrhea [37,38,39,40,41,42]. These results are in agreement with the findings of Sun W et al. (2022) which showed that the increase of the genera in Enterococcus and Enterococcaceaewere also the most notable features in heart failure patients [43]. Increased abundance ofcertain pathogenic microbes (e.g., *Helicobacter pylori* [44], *Clostridium* spp. [45], *Enterococcus faecalis* [46], and *Staphylococcus* spp. [47]) were associated with leaky gut and cardiovascular diseases, which correlates with the obtained data.

## 5. Conclusions

The present study was conducted to investigate the composition of the gut microbiota in patients with atherosclerotic cardiovascular disease and certain heart failure syndromes and to compare these results with the microbiota of individuals without those diseases (control group). When choosing the control group for comparison, the main characteristics of the original group (age, BMI, gender) were taken into account; however, it was not possible to exclude all persons who were not fully applicable to the target group. For this reason, the influence of other factors that could have affected the composition of the microbiota and changed the results of the study could not be excluded. Despite this, in the case of real-time qPCR quantification, the choice of extraction method is likely to have a much greater impact on the results, which can significantly affect the number of some of the estimated OTUs. The major difference between the two extraction kits is the bead beating phase, which can have a significant impact on the determined relative bacterial abundances in the purified samples.

In the case of the NGS study, it was shown that the increased abundance of family Erysipelotrichaceae strictly correlated with all three disease groups (HFpEF, HFrEF, ASCVD); besides, it was the the only one with a statistically significant factor for the HFpEF group. Additionally, the increased abundances of families Streptococcaceae and Sutterellaceae were the most significant factors for the ACVD group, while for the HFrEF group, the most significant factors were increased abundances of families Streptococcaceae, Erysipelotrichaceae, Lactobacillaceae, and Clostridiaceae and a decreased abundance of the family Acidaminococcaceae.

The MALDI-TOF data indirectly confirmed the results for the family Streptococcaceae for the HFrEF group. Still, it is important to note that sometimes significant differences between MALDI-TOF and other methods’ results were noted: for instance, for the HFpEF group, no overlapping conclusions were observed. This can be explained by the difficulty of cultivating the majority of gut bacteria: bacterial cultures can detect only the living organisms, while NGS captures total DNA.

Most of the taxa in this qPCR study showed insignificant correlations with the disease groups in accordance with the NGS data, and, considering the fact that none of the observed differences in bacterial abundance during the Mann–Whitney U test corresponded to both extraction methods, it should be concluded that there was no association of the selected OTUs (families Enterobacteriaceae, Christensenellaceae; genera *Bacteroides* sp., *Bifidobacterium* sp., *Odoribacter* sp., *Oscillibacter* sp.; species *Enterococcus faecalis*, *Faecalibacterium prausnitzii*) with either coronary atherosclerosis or heart failure syndromes. The only exception is the family Lactobacillaceae which was confirmed independently with qPCR and NGS—and also indirectly with bacterial culture—and thus may be proposed as a taxon that positively correlated with atherosclerosis.

Since microbiota composition can be profoundly affected by several factors that are not easy controlled, such as diet, the interpretation of limited data should be conducted with caution. Further investigation is required to confirm these, and to find other, reliable connections between gut microbiota and CVD. The similarities and differences in the conclusions obtained by the three methods of this study demonstrate the need for a comprehensive approach to the analysis of microbiota.

## Figures and Tables

**Table 1:** The main characteristics of patients.

Characteristics	HFpEF (n = 59)	HFrEF (n = 50)	ASCVD (n = 100)	Control Group (n = 50)
Age, y	67.0 [61.0; 71.0]	68.5 [61.0; 73.0]	66.0 [59.0; 71.0]	57.5 [48.0; 63.0]
Sex, male, n (%)	30 (50.8%)	46 (92%)	66 (66.0%)	22 (44%)
BMI, kg/m^2^	31.2 [27.4; 33.6]	28.9 [25.8; 32.0]	29.1 [27.3; 31.2]	26.3 [22.9; 29.7]
NYHA functional class, n (%)			-	-
I	42 (71.1%)	9 (18.0%)		
II	17 (28.9%)	36 (72.0%)		
III	0	5 (10.0%)		
IV	0	0		
Hypertensive, n (%)	58 (98.3%)	45 (90.0%)	92 (92.0%)	27 (54%)
Type 2-diabetes, n (%)	0 (0%)	38 (76.0%)	6 (6.0%)	0 (0%)
Dyslipidemia, n (%)	34 (57.7%)	10 (20.0%)	80 (80.0%)	21 (42%)
AF, n (%)	29 (49.1%)	24 (48.0%)	24 (24.0%)	0 (0%)
CAD, n (%)	3 (5.1%)	49 (98.0%)	92 (92.0%)	0 (0%)
Revascularization, n (%)	2 (3.3%)	43 (86.0%)	84 (84.0%)	0 (0%)
Catheter ablation, n (%)	10 (16. 9%)	0 (0%)	5 (5.0%)	0(0%)
GFR, mL/min/1.73 m^2^	78.0 [68.0; 88.0]	71.5 [54.0; 85.0]	79.0 [70.5; 90.5]	86.2 [77.6; 96.5]
NT-proBNP, pg/mL	178.0 [136.0; 295.0]	-	-	-
Echocardiography				
LVEDD, mm	49.0 [48.0; 52.0]	65.0 [60.0; 70.0]	52.0 [48.0; 53.0]	47.0 [46.0; 49.0]
LV EF, %	64.0 [59.5; 67.0]	34.0 [30.0; 40.0]	62.0 [58.0; 67.0]	63.0 [60.0; 66.0]
LV Mass Index, g/m^2^	115.0 [100.5; 126.0]	255.5 [210.0; 320.0]	110.0 [90.0; 124.5]	87.5 [76.0;97.0]
LA Volume Index, mL/m^2^	37.0 [35.0; 43.5]	58.0 [50; 70]	39.7 [35.0; 43.0]	26.0 [22.0; 31.0]
E, sm/s	78.0 [67.0; 86.5]	71.0 [59.0; 85.0]	67.5 [52.0; 81.0]	65.0 [61.0; 71.0]
A, sm/s	78.0 [65.0; 86.5]	63.5 [50.0; 85.0]	75.0 [64.0; 89.0]	54.0 [51.0; 65.0]
e’, sm/s	6.0 [5.0; 6.4]	-	-	9.1 [8.6; 11.0]
E/A	1.05 [0.82; 1.24]	0.86 [0.7; 1.59]	0.79 [0.73; 0.98]	1.2 [1.1; 1.35]
E/e’	13.0 [13.0; 14.0]	-	-	6.9 [6.0; 7.2]
Medications				
RAAS inhibitors, n (%)	56 (94.9%)	45 (90.0%)	85 (85.0%)	25 (50.0%)
Beta-clockers, n (%)	51 (86.4%)	45 (90.0%)	66 (66.0%)	10 (20.0%)
Diuretics, n (%)	49 (83%)	42 (84.0%)	28 (28.0%)	8 (16.0%)
Anticoagulants, n (%)	22 (37.3%)	22 (44.0%)	18 (18.0%)	0 (0%)
Antiplatelet agents, n (%)	11 (18.6%)	32 (64.0%)	88 (88.0%)	7 (14.0%)
Statins, n (%)	34 (57.6%)	38 (76.0%)	86 (86.0%)	17 (34.0%)
Proton pump inhibitors, n (%)	17 (28.8%)	16 (32.0%)	43 (43.0%)	12 (24.0%)

A—late (atrial) diastolic transmitral flow velocity, ASCVD—atherosclerotic cardiovascular disease, AF—atrial fibrillation, BMI—body mass index, E—early diastolic transmitral flow velocity, E/A—early to atrial filling velocity ratio; e′—early diastolic mitral annular velocity, E/e’ = early mitral inflow velocity to early diastolic mitral annulus velocity ratio; HFpEF = heart failure with preserved ejection fraction; HFrEF = heart failure with reduced ejection fraction; LA—left atrium/atrial, LVEDD—left ventricular end-diastolic diameter, LV EV—left ventricular ejection fraction, NYHA: the New York Heart Association, RAAS inhibitors—renin-angiotensin-aldosterone system inhibitors.

**Table 2:** Baseline characteristics of patients for MALDI-TOF-MS analysis.

Characteristics	HFpEF (n = 37)	Control Group (n = 36)	HFrEF (n = 41)	Control Group (n = 42)	ASCVD (n = 69)	Control Group (n = 43)
Age, y Average ± SD [min; max]	66.0 ± 8.9 [41; 79]	53.4 ± 12.7 [25; 76]	70.0 ± 8.6 [41; 80]	52.2 ± 13.8 [25; 76]	66.5 ± 8.5 [40; 80]	52.3 ± 13.7 [25; 76]
Sex, male, n (%)	21 (56.8%)	8 (22.2%)	37 (90.2%)	9 (21.4%)	46 (66.7%)	9 (20.9%)
BMI, kg/m^2^ Average ± SD [min; max]	28.6 ± 3.5 [21.5; 32.8]	25.9 ± 3.7 [20.5; 33.9]	27.8 ± 3.3 [20.4; 33.1]	25.0 ± 4.1 [18.3; 33.9]	27.8 ± 2.5 [20.5; 31.8]	24.9 ± 4.1 [18.2; 33.9]

**Table 3:** Baseline characteristics of patients for NGS analysis.

Characteristics	HFpEF (n = 16)	HFrEF (n = 18)	ASCVD (n = 77)	Control Group (n = 17)
Age, y Average ± SD [min; max]	65.1 ± 11.1 [41; 79]	65.6 ± 6.6 [51; 74]	65.7 ± 9.1 [40; 80]	50.9 ± 13.5 [31; 76]
Sex, male, n (%)	12 (75.0%)	17 (94.4%)	51 (66.2%)	4 (23.5%)
BMI, kg/m^2^ Average ± SD [min; max]	28.4 ± 4.5 [21.5; 34.6]	27.8 ± 2.0 [24.2; 31.1]	28.6 ± 2.9 [20.5; 33.4]	25.6 ± 4.0 [19.8; 33.9]

**Table 4:** Baseline characteristics of patients for qPCR analysis, FS extraction method.

Characteristics	HFpEF (n = 13)	HFrEF (n = 20)	ASCVD (n = 37)	Control Group (n = 28)
Age, years Average ± SD [min; max]	68.0 ± 8.4 [54; 80]	68.7 ± 6.5 [56; 80]	66.2 ± 9.4 [40; 80]	54.3.2 ± 9.3 [41; 74]
Sex, male, n (%)	6 (46.1%)	17 (85%)	26 (70.3%)	19 (67.8%)
BMI, kg/m^2^ Average ± SD [min; max]	30.5 ± 3.5 [23.9; 34.6]	28.9 ± 3.9 [21.8; 34.7]	28.6 ± 3.7 [19.8; 36.3]	28.3 ± 3.5 [21.9; 34.5]

**Table 5:** Baseline characteristics of patients for qPCR analysis, PF extraction method.

Characteristics	HFpEF (n = 16)	HFrEF (n = 25)	ASCVD (n = 37)	Control Group (n = 21)
Age, y Average ± SD [min; max]	68.0 ± 9.1 [49; 80]	70.0 ± 6.6 [55; 80]	63.7 ± 8.8 [55; 80]	56.5 ± 8.6 [44; 74]
Sex, male, n (%)	8 (50%)	22 (88%)	26 (70.3%)	13 (61.9%)
BMI, kg/m^2^ Average ± SD [min; max]	29.5 ± 3.8 [22.1; 34.6]	28.3 ± 3.9 [20.4; 34.7]	29.8 ± 2.7 [25.8; 34.7]	28.2 ± 4.3 [21.1; 34.9]

**Table 6:** Results of MALDI-TOF MS analysis. Arrows indicate increase (↑) or decrease (↓) in the patients group.

List of Bacterial Species	Relative Abundance	Effect Size	Adjusted *p*-Value
1. Atherosclerotic cardiovascular diseases			
*Enterococcus faecium*	↓	0.23	<0.001
*Enterobacter cloacae*	↓	0.2	<0.001
*Bifidobacterium bifidum*	↓	0.18	<0.001
*Enterococcus faecalis*	↓	0.17	<0.001
*Escherichia coli*	↓	0.12	<0.01
*Citrobacter freundii*	↓	0.1	<0.05
*Pediococcus acidilactici*	↓	0.1	<0.05
2. Heart failure with preserved ejection fraction			
*Enterococcus faecium*	↓	0.45	<0.001
*Enterococcus faecalis*	↓	0.19	<0.01
*Bacteroides fragilis*	↓	0.12	<0.05
3. Heart failure with reduced ejection fraction			
*Streptococcus parasanguinis*	↑	0.24	<0.001
*Streptococcus sanguinus*	↑	0.21	<0.001
*Candida albicans*	↑	0.11	<0.05
*Enterococcus faecalis*	↓	0.37	<0.001
*Bifidobacterium bifidum*	↓	0.18	<0.01
*Bacteroides fragilis*	↓	0.14	<0.05
*Enterococcus faecium*	↓	0.13	<0.05

**Table 7:** Results of 16S RNA sequencing analysis. Arrows indicate increase (↑) or decrease (↓) in the patients group.

Families	Relative Abundance	Effect Size, RDP/SILVA	Adjusted *p*-Value
1. Atherosclerotic cardiovascular diseases			
Erysipelotrichaceae	↑	0.28/0.18	<0.001
Streptococcaceae	↑	0.2/0.2	<0.001
Clostridiaceae	↑	0.14/0.14	<0.01
Peptostreptococcaceae	↑	0.14/0.14	<0.01
Lactobacillaceae	↑	0.12/0.12	<0.01
Bifidobacteriaceae	↑	0.11/0.11	<0.01
Sutterellaceae	↓	0.28/0.35	<0.001
Acidaminococcaceae	↓	0.17/0.17	<0.005
Odoribacteraceae	↓	0.13/0.13	<0.001
2. Heart failure with preserved ejection fraction			
Erysipelotrichaceae	↑	0.28/0.11	<0.05
3. Heart failure with reduced ejection fraction			
Streptococcaceae	↑	0.48/0.48	<0.01
Erysipelotrichaceae	↑	0.5/0.34	<0.05
Lactobacillaceae	↑	0.45/0.39	<0.01
Clostridiaceae	↑	0.38/0.38	<0.01
Methanobacteriaceae	↑	0.28/0.28	<0.05
Bifidobacteriaceae	↑	0.28/0.28	<0.05
Eggerthellaceae	↑	0.25/0.36	<0.05
Enterococcaceae	↑	0.25/0.25	<0.05
Acidaminococcaceae	↓	0.43/0.43	<0.01
Barnesiellaceae	↓	0.25/0.36	<0.05
Odoribacteraceae	↓	0.27/.027	<0.05

## Data Availability

The data presented in this study are available on request from the corresponding author.

## Supplementary Materials

The following supporting information can be downloaded at: https://www.mdpi.com/article/10.3390/biomedicines10112762/s1. Figure S1: Influence of the extraction method on qPCR results, PCA plots for all investigated groups of patients and control. Figure S2: PCA and LDA analysis for qPCR results: comparison of control and disease groups for both extraction methods, Power Fecal and Fast Stool. Table S1: Primer sets used in the qPCR study.
