# Supplementary material for: Diversities in the Gut Microbial Patterns in Patients with Atherosclerotic Cardiovascular Diseases and Certain Heart Failure Phenotypes
Source: Biomedicines. 2022 Oct 31;10(11):2762. doi: 10.3390/biomedicines10112762 (PMC9687836; doi:10.3390/biomedicines10112762)
Supplement: Supplementary file 1 [file biomedicines-10-02762-s001.zip › biomedicines-1970969 sup update .pdf]

Supplementary materials

Table S1. Primer sets used in the qPCR study.

Target organism	Primer set	Sequence (5′ to 3′)
Total bacteria amount	515F	GTGCCAGCAGCCGCGGTAA
	806R	GACTACCAGGGTATCTAATCC
	Uni probe Fam/BHQ1	TMTCTRMGCATTYCACCGCTAC
Bacteroides sp.	Bac F	ATTGGGTTTAAAGGGAGCGT
	Bac R	CTACACCACGAATTCCGCCT
	Bac P Fam/BHQ1	TCAGTTGTGAAAGTTTGCGGCTCAAC
Enterobacteriaceae	Ent-ceae F	TCCCCGGGCTCAACCTG
	Ent-ceae R	CCAARTCGACATCGTTTACG
	Ent-ceae P Fam/BHQ1	CTCTACRAGACTCWAGCYTGCCA
Lactobacillaceae	Lac-ceae F	CAGCAGTAGGGAATCTTCCA
	Lac-ceae R	AGTTTCCGATGCRVTTC
	Lac-ceae P Fam/BHQ1	TGATGGAGCAACRCCGCGTG
Christensenellaceae	Christ F	ACCGCATGAGACCACGAAACCG
	Christ R	TCCCATAGGACAAAGGTTTAC
	Christ P Fam/BHQ1	TCGATGCAGGATGGGCTCGCG
Bifidobacterium sp.	Bif F	CGAACGGGTGAGTAATGCGT
	Bif R	TGATAGGACGCGACCCCATC
	Bif P Fam/BHQ1	CCGGAATAGCTCCTGGAAACGGGT
Odoribacter sp.	Odor F	GCCCATGGAAACGTGGAT
	Odor R	CAGTCTTCCCTCACGCGA
	Odor P Fam/BHQ1	AGGGGTTCTGAGAGGAAGGTC
Oscillibacter sp.	Oscill F	TTAACTGCGGCACGCAG
	Oscill R	GTGTTAACTGCGGCACG
	Oscill P Fam/BHQ1	AGGTGTGCGGGGACTGACC
Enterococcus faecalis	Ent fae F	GCAAGTCGAACGCTTCTTTC
	Ent fae R	GCCTTTCACTCTTATGCCATG

	Ent fae P Fam/BHQ1	CATAAACTGTTATGCGGTATTAGCAC C
Faecalibacterium prausnitzii	Fae prau F	AAGATAATGACGGTACYCAACAAG
	Fae prau R	CACTTCCAACTTGTCTTCCCGC
	Fae prau P Fam/BHQ1	CAACGCTTGTGACCTACGTTTTAC

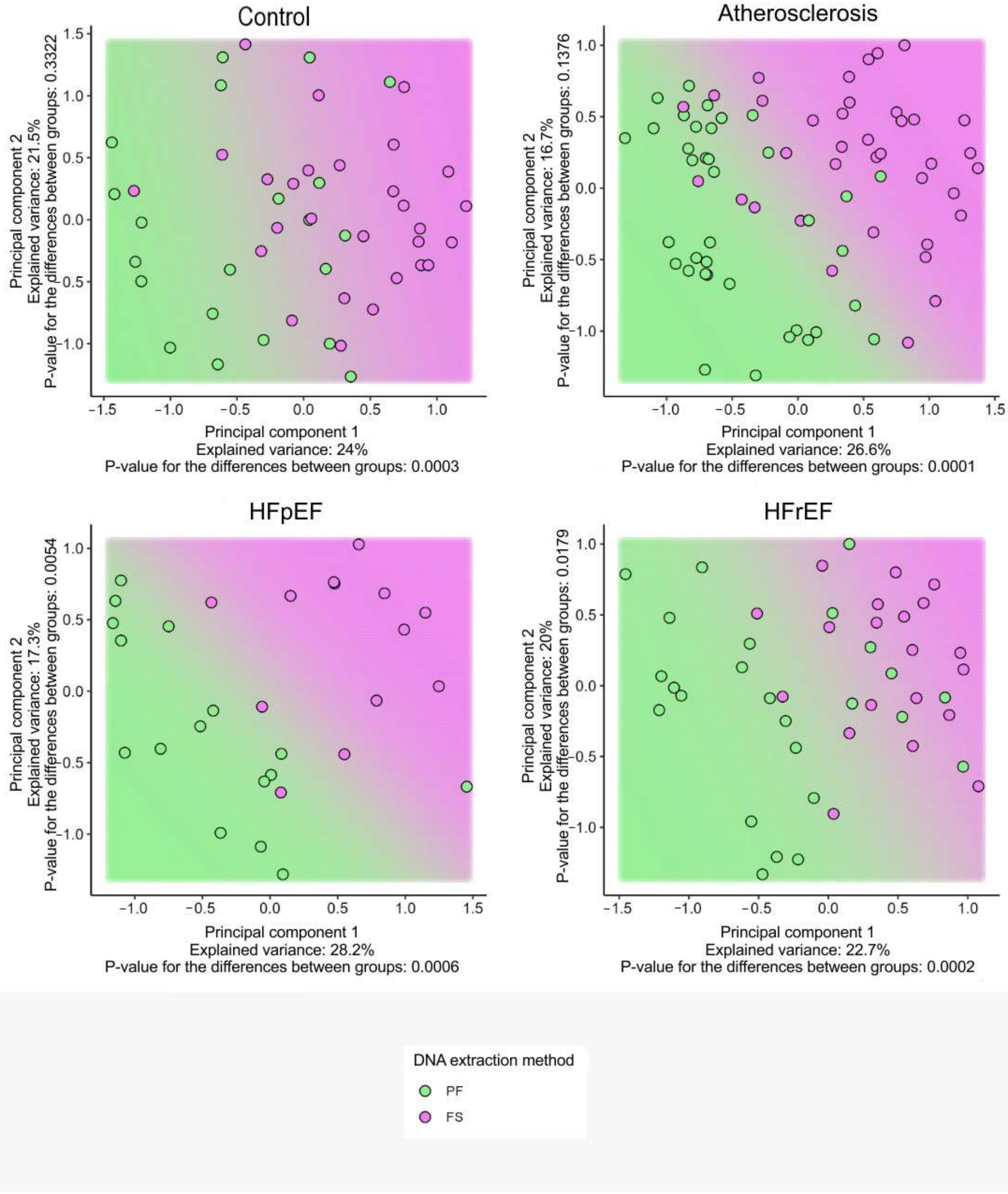

Figure S1. Influence of the extraction method on qPCR results, PCA plots for all investigated groups of patients and control. All individual OTU (bacterial) concentrations were subjected to PCA and the first two principal components, PC1 and PC2, were plotted. Green circles indicate PF method; violet dots – FS method. Profiles of each method in the plots indicate that the choice of DNA ex-traction method affects the results of qPCR study.

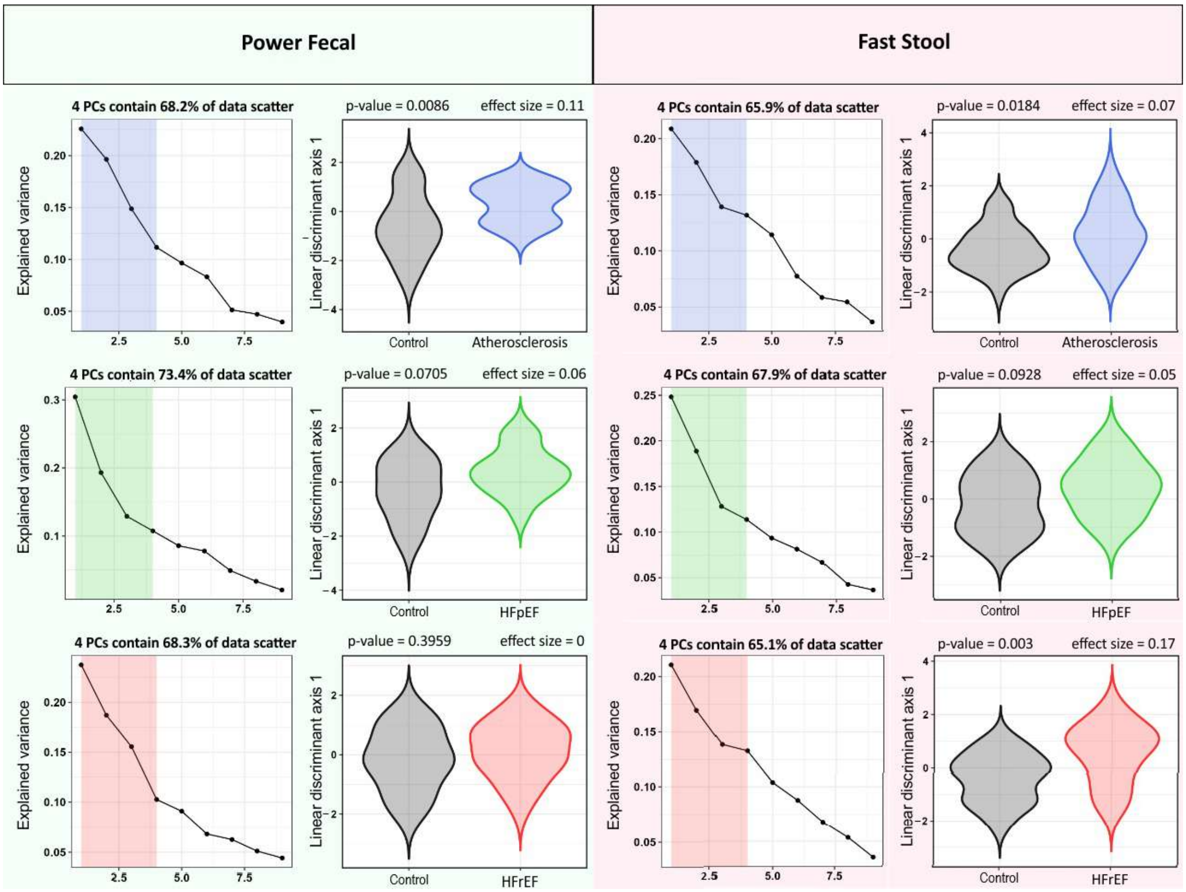

Figure S2. PCA and LDA analysis for qPCR results: comparison of control and disease groups for both extraction methods, Power Fecal and Fast Stool. Results of PCA data processing, the established correlation between the best discriminant axis and initial indicators, the distribution of the ob-served group values across the best discriminant axis. Blue: graphs for ASCVD; green: graphs for HFpEF; red: graphs for HFrEF. Despite the absence of clear differences between the samples of patients and healthy individuals for each particular taxon, the combination all PCR taxa for principal component method and linear discriminant analysis managed to reliably separate healthy control from patients with atherosclerosis for both extraction methods.
